# Economic Games on the Internet: The Effect of $1 Stakes

**DOI:** 10.1371/journal.pone.0031461

**Published:** 2012-02-21

**Authors:** Ofra Amir, David G. Rand, Ya'akov Kobi Gal

**Affiliations:** 1 Department of Information Systems Engineering, Ben-Gurion University of the Negev, Be'er Sheva, Israel; 2 Program for Evolutionary Dynamics, Harvard University, Cambridge, Massachusetts, United States of America; 3 School of Engineering and Applied Sciences, Harvard University, Cambridge, Massachusetts, United States of America; University of Maribor, Slovenia

## Abstract

Online labor markets such as Amazon Mechanical Turk (MTurk) offer an unprecedented opportunity to run economic game experiments quickly and inexpensively. Using Mturk, we recruited 756 subjects and examined their behavior in four canonical economic games, with two payoff conditions each: a stakes condition, in which subjects' earnings were based on the outcome of the game (maximum earnings of $1); and a no-stakes condition, in which subjects' earnings are unaffected by the outcome of the game. Our results demonstrate that economic game experiments run on MTurk are comparable to those run in laboratory settings, even when using very low stakes.

## Introduction

Online labor markets such as Amazon Mechanical Turk (MTurk) are internet marketplaces in which people can complete short tasks (typically 5 minutes or less) in exchange for small amounts of money (typically $1 or less). MTurk is becoming increasingly popular as a platform for conducting experiments across the social sciences [1]–[7]. In particular, MTurk offers an unprecedented opportunity to run incentivized economic game experiments quickly and inexpensively. Recent work has replicated classical findings such as framing and priming on MTurk [8]–[10], found a high level of test-retest reliability on Mturk [10]–[12], and shown quantitative agreement in behavior between MTurk and the physical laboratory [6], [8]. Yet concerns remain regarding the low stakes typically used in MTurk experiments.

In this study, we directly examine the effect of such stakes by comparing un-incentivized play with play involving typical MTurk sized stakes (up to $1) in four canonical economic games - the dictator game, ultimatum game, trust game and public goods game. Our results are consistent with previous research conducted in the physical laboratory using an order of magnitude higher stakes.

Prior work on the dictator game found that subjects became significantly less generous when going from no stakes to low stakes [13], but that going from low stakes to high stakes did not affect donations [13], [14]. Consistent with these results, we find that the average donation on MTurk decreases from 44% with no stakes to 33% with $1 stakes.

Prior work on the ultimatum game has found that adding stakes does not affect the average proposal but may increase the variance in proposals [13], [15], while the results for responder behavior are more mixed, with one study finding no effect [13] and another finding a significant decrease in rejection rates [15]. It has also been found that increasing from low to high stakes has little effect on either proposals or rejection rates, unless the stakes are extremely large [13]–[17]. Our results when comparing no stakes with $1 stakes on MTurk are broadly consistent with these previous findings. In particular, we see no difference in Player 1 proposals, or the minimal amount accepted by Player 2 s when excluding ‘inconsistent’ players (people who accepted some offer 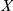 while also rejecting one or more offers greater than 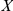). However we do find that adding stakes decreases the fraction of such inconsistent Player 2 s, and decreases rejection rates of some Player 1 offers when including inconsistent Player 2 s.

There has been less study of the role of stakes in other social dilemma games. To our knowledge, comparisons between no stakes and low stakes have not been performed in the public goods game or the trust game. Considering the increase of stake size, Kocher, Martinsson and Visser [18] found no significant difference in subjects' contributions in the public goods game when going from low to high stakes, and Johansson-Stenman, Mahmud and Martinsson [19] found that in the trust game, the amount sent by investors decreased when using very high stakes but the fraction returned by trustees was not affected by the changes in stakes. We find no difference in cooperation in the public goods game or trust or trustworthiness in the trust game when comparing no stakes with $1 stakes on MTurk.

## Materials and Methods

This research was approved by the committee on the use of human subjects in research of Harvard University, application number F17468-103. Informed consent was obtained from all subjects.

We recruited 756 subjects using MTurk and randomly assigned each subject to play one of four canonical games - the dictator game, ultimatum game, trust game and public goods game - either with or without stakes. In all eight conditions, subjects received a $0.40 show up fee. In the four stakes conditions, subjects had the opportunity to earn up to an additional $1.00 based on their score in the game (at an exchange rate of 1 point = 1 cent). In the four no-stakes conditions, subjects were informed of the outcome of the game, but the score in the game did not affect subjects' earnings. In all conditions, subjects had to complete a series of comprehension questions about the rules of the game and their compensation, and only subjects that answered all questions correctly were allowed to participate. We now explain the implementation details of each of the four games.

In the Dictator game (DG), Player 1 (the dictator) chose an amount 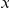 (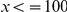) to transfer to Player 2, resulting in Player 1 receiving a score of 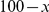 and Player 2 receiving a score of 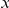.

In the Ultimatum Game (UG), Player 1 (the proposer) chose an amount 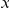 (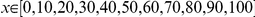) to offer to Player 2 (the responder). Player 2 could then accept, resulting in Player 1 receiving a score of 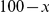 and Player 2 receiving a score of 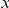; or reject, resulting in both players receiving a score of 0. We used the strategy method to elicit Player 2 decisions (i.e., Player 2 indicated whether she would accept or reject each possible Player 1 offer). For each Player 2 we then calculated her Minimum Acceptable Offer (MAO) as the smallest offer she was willing to accept. As in the physical lab, some subjects were ‘inconsistent’ in that they were willing to accept some of the lower offers, but rejected higher offers (that is, they did not have a threshold for acceptance) [20]. When calculating MAOs, we did not include such inconsistent players. We also examined how the addition of stakes changed the fraction of inconsistent players, as well as the rejection rates for each possible Player 1 offer when including all Player 2 s (consistent and inconsistent).

In the Trust Game (TG), Player 1 (the investor) chose an amount 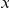 (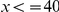) to transfer to Player 2 (the trustee). The transferred amount was multiplied by 3 and given to the trustee, who then chose a fraction 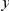 (where 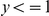) to return to Player 1. As a result, Player 1 received a score of 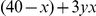 and Player 2 received a score of 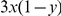. We used the strategy method to elicit Player 2 decisions (i.e., Player 2 indicated the fraction she would return for each possible Player 1 transfer).

In the Public Goods Game (PGG), four players each received an initial endowment of 40 units, and simultaneously choose an amount 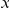 (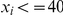) to contribute to a public pool. The total amount in the pot was multiplied by 2 and then divided equally by all group members. As a result, player i received the score 
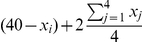
.

In the DG, UG and TG experiments, each subject played both roles, first making a decision as Player 1, and then making a decision as Player 2. Subjects were not informed that they would subsequently play as Player 2 when making their Player 1 decisions. Unless otherwise noted, all statistical tests use the Wilcoxon Rank-sum test.

## Results and Discussion

**Figure 1:**
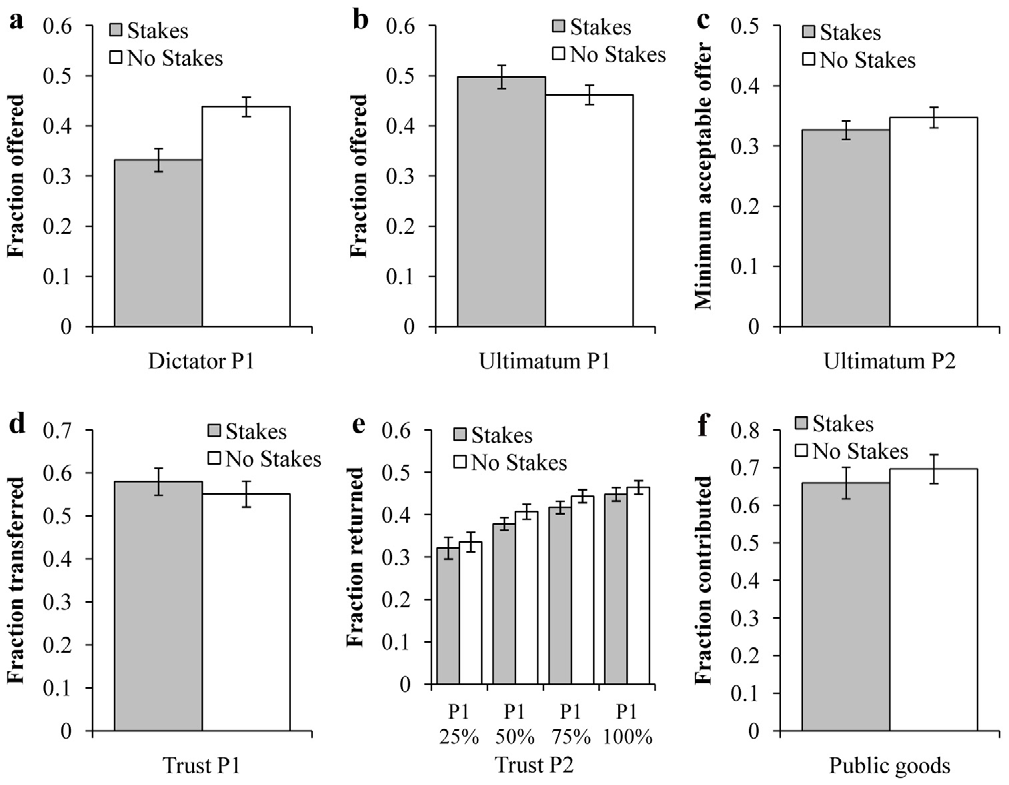
The effect of stakes on average behavior across games.

As can be seen in Figure 1, introducing stakes altered the distribution of offers in the DG, significantly reducing the average offer (no-stakes = 43.8%, stakes = 33.2%, 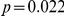). In the UG, we found a marginally significant positive effect of stakes on Player 1 proposals (no-stakes = 46.1%, stakes = 49.7%, 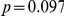). Given the small effect size and borderline significant p-value, we conclude that stakes have little effect on P1 offers in the UG. We also find no significant effect on Player 2 MAOs in the UG (excluding inconsistent players) (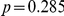). However, we do find a significantly higher proportion of inconsistent Player 2′s in the no-stakes condition compared to the stakes condition (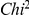 test, 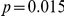). As a result, we also find a significant effect of stakes on Player 2 rejection rates for some Player 1 offers in the UG when including inconsistent players (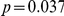 for the 30% offer, and 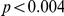 for all offers above 60%). There was no significant effect of stakes on transfers in the TG (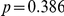), back-transfers in the TG (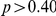 for all possible Player 1 transfers), and contributions in the PGG (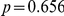). We also test whether the variance in behavior differs between the stakes and no-stakes conditions using Levene's F-test. Consistent with our results above, we find that the variance in DG donations is significantly smaller in the stakes condition compared to the no-stakes condition (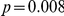), but that adding stakes did not have an effect on the variance of offers (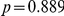) and MAOs in the UG (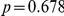), transfers (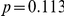) and back-transfers in the TG (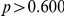 for back-transfers on all Player 1 transfers, except for the transfer of 25% where the variance of Player 2 back-transfers in the stakes condition was marginally higher, 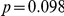), and contributions in the PGG (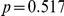).

Furthermore, we find that the average behavior on MTurk is in line with behavior observed previously in the physical laboratory with higher stakes. The average donation of 33.2% in our $1 stakes DG is close to the average donation of 28.4% aggregated over more than 616 DG treatments as reviewed in a recent meta-analysis [21].

Since there was little difference in behavior between the stakes and no-stakes conditions in the UG, TG and PGG, we compare the aggregated averages from both conditions in these games to prior work. Considering the UG, it has been shown that using the strategy method significantly affects behavior [22], and to our knowledge no meta-analyses exist which focus on strategy-method UGs. Therefore, we examine behavior in various previous UG experiments that used the strategy method [23]–[29], and compare the range of outcomes to what we observe in our data. The average Player 1 offer of 48.1% in our experiment is within the range of behavior observed in those studies (35.4%–48.4%), as is our average Player 2 MAO of 33.7% (compared to the range of previous MAOs of 19.2%–36.0%).

Turning now to the TG, we find that the average percentage sent by Player 1 in our experiment (56.6%) is quite close to the average value of 50.9% reported in a recent trust game meta-analysis aggregating over approximately 80 experiments [30]. The fraction returned by Player 2 of 40.1% in our experiment is also close to the average returned fraction of 36.5% from the same meta-analysis.

For the public goods game, it is important to compare our results to those obtained in previous experiments using the same Marginal Per-Capita Return value (MPCR = 0.5 in our study). In the absence of a meta-analysis that breaks contributions down by MPCR, we compare the average contribution level in our experiment to the range of average contributions observed in various previous studies using the same MPCR [31]–[36]. The average fraction of the endowment contributed to the public good in our study of 67.7% is within the range observed in these studies (40%–70.4%).

To conclude, we have assessed the effect of $1 stakes compared to no stakes in economic games run in the online labor market Amazon Mechanical Turk. The results are generally consistent with what is observed in the physical laboratory, both in terms of the effect of adding stakes, and the average behavior in the stakes conditions. These experiments help alleviate concerns about the validity of economic game experiments conducted on MTurk and demonstrate the applicability of this framework for conducting large scale scientific studies.
